# Supplementary material for: Mitochondrial genomes of two parasitic Cuscuta species lack clear evidence of horizontal gene transfer and retain unusually fragmented ccmFC genes
Source: BMC Genomics. 2021 Nov 12;22:816. doi: 10.1186/s12864-021-08105-z (PMC8588681; doi:10.1186/s12864-021-08105-z)
Supplement: Supplementary file 1 — Additional file 1: Fig. S1. Chloroplast genome of Cuscuta australis. Transcription is counterclockwise around the outside of the circle and clockwise inside. The grey inner graph is GC content. Abbreviations: LSC = large single copy region, SSC = small single copy region, IRA/B = inverted repeat A/B. Fig. S2. Chloroplast genome of Cuscuta campestris. Transcription is counterclockwise around the outside of the circle and clockwise inside. The grey inner graph is GC content. Abbreviations: LSC = large single copy region, SSC = small single copy region, IRA/B = inverted repeat A/B. Fig. S3. Dispersed repeats in the mitogenomes of Cuscuta australis (left) and C. campestris (right). Generated with Circoletto (http://tools.bat.infspire.org/circoletto/). Fig. S4. Read depth for mitogenome assemblies of Cuscuta australis (top) and C. campestris (bottom). Short read depth is in black while long read depth is in green. Breakpoints between contigs in C. australis are blue lines. Minimum and mean read depths are indicated, with colours of text matching respective read depth. Fig. S5. Edited sites in ccmFC from Cuscuta australis based on mapped transcriptomic reads and predicted by PREP-MT. Exons are indicated above the mapped reads, with circles corresponding to panels above showing the mismatches in more detail. Positions along the alignment are numbered, with positions not predicted by PREP-MT circled in blue, and those predicted but not observed by mapping circled in red. The table shows details of the results from PREP-MT. Table S1. Repeats recombination analysis for Cuscuta australis. The analysis is based on non-overlapping repeats > 500 bp and > 90% identity (blastn evalue 1e-10), and mapping long reads to those repeats. Table S2. Repeats recombination analysis for Cuscuta campestris. The analysis is based on non-overlapping repeats > 500 bp and > 90% identity (blastn evalue 1e-10), and mapping long reads to those repeats. [file 12864_2021_8105_MOESM1_ESM.pdf]

Additional file 1: supplementary Figs. S1–S5, Tables S1 & S2

Mitochondrial genomes of two parasitic *Cuscuta* species lack clear evidence of horizontal gene transfer and retain unusually fragmented *ccmF*$_C$ genes

Anderson, Benjamin M; Krause, Kirsten; and Petersen, Gitte

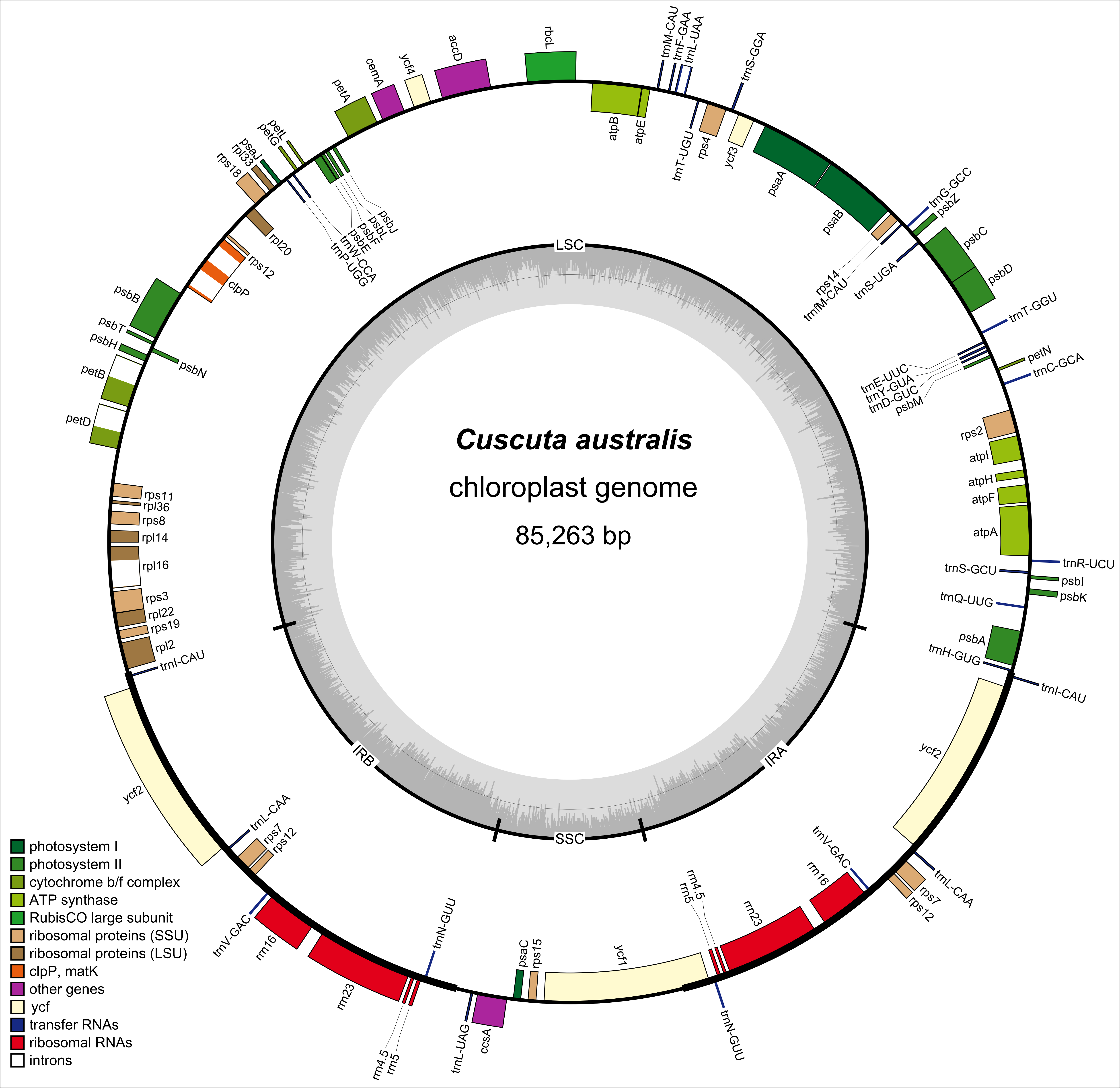
Cuscuta australis
chloroplast genome
85,263 bp
LSC
IRB
IRA
SSC
rbcL
accD
ycf4
cemA
petA
petL
petG
psaJ
rpl33
rps18
psbJ
psbL
psbF
psbE
trnW-CCA
trnP-UGG
rpl20
rps12
clpP
psbB
psbT
psbH
psbN
petB
petD
rps11
rpl36
rps8
rpl14
rpl16
rps3
rpl22
rps19
rpl2
trnI-CAU
ycf2
trnL-CAA
rps7
rps12
trnV-GAC
rrn16
rrn23
rrn4.5
rrn5
trnN-GUU
trnL-UAG
ccsA
psaC
rps15
ycf1
rrn4.5
rrn5
rrn23
trnN-GUU
rrn16
trnV-GAC
rps7
rps12
trnL-CAA
ycf2
trnI-CAU
trnH-GUG
psbA
trnQ-UUG
psbK
psbI
trnS-GCU
trnR-UCU
atpA
atpF
atpH
atpI
rps2
trnE-UUC
trnY-GUA
trnD-GUC
psbM
petN
trnC-GCA
trnT-GGU
psbD
psbC
trnS-UGA
rps14
trnfM-CAU
trnG-GCC
psbZ
psaB
psaA
ycf3
rps4
trnT-UGU
trnS-GGA
trnM-CAU
trnF-GAA
trnL-UAA
atpB
atpE
photosystem I
photosystem II
cytochrome b/f complex
ATP synthase
RubisCO large subunit
ribosomal proteins (SSU)
ribosomal proteins (LSU)
clpP, matK
other genes
ycf
transfer RNAs
ribosomal RNAs
introns

Fig. S1 (previous page). Chloroplast genome of *Cuscuta australis*. Transcription is counterclockwise around the outside of the circle and clockwise inside. The grey inner graph is GC content. Abbreviations: LSC = large single copy region, SSC = small single copy region, IRA/B = inverted repeat A/B.

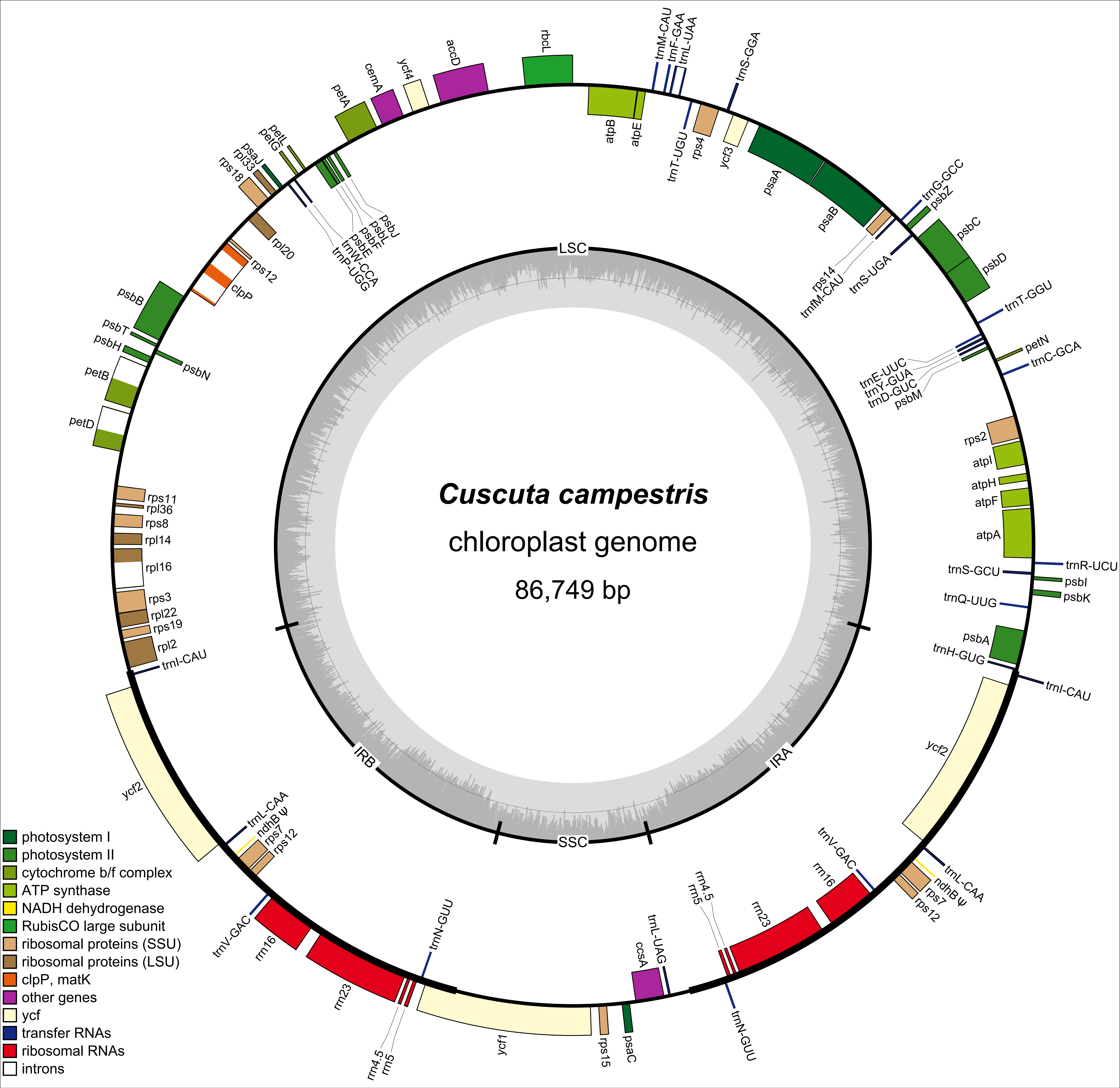
Cuscuta campestris
chloroplast genome
86,749 bp
LSC
IRA
SSC
IRB
rbcL
accD
ycf4
cemA
petA
trnM-CAU
trnF-GAA
trnL-UAA
trnS-GGA
atpB
atpE
trnT-UGU
rps4
ycf3
psaA
psaB
trnG-GCC
psbZ
psbC
psbD
rps14
trnfM-CAU
trnS-UGA
trnT-GGU
petN
trnC-GCA
trnE-UUC
trnY-GUA
trnD-GUC
psbM
rps2
atpI
atpH
atpF
atpA
trnR-UCU
trnS-GCU
psbI
psbK
trnQ-UUG
psbA
trnH-GUG
trnI-CAU
ycf2
trnL-CAA
ndhB ψ
rps7
rps12
trnV-GAC
rrn16
rrn23
rrn4.5
rrn5
trnN-GUU
trnL-UAG
ccsA
psaC
rps15
ycf1
petL
petG
psaJ
rpl33
rps18
rpl20
rps12
clpP
psbJ
psbL
psbF
psbE
trnW-CCA
trnP-UGG
psbB
psbT
psbH
psbN
petB
petD
rps11
rpl36
rps8
rpl14
rpl16
rps3
rpl22
rps19
rpl2
trnI-CAU
photosystem I
photosystem II
cytochrome b/f complex
ATP synthase
NADH dehydrogenase
RubisCO large subunit
ribosomal proteins (SSU)
ribosomal proteins (LSU)
clpP, matK
other genes
ycf
transfer RNAs
ribosomal RNAs
introns

Fig. S2 (previous page). Chloroplast genome of *Cuscuta campestris*. Transcription is counterclockwise around the outside of the circle and clockwise inside. The grey inner graph is GC content. Abbreviations: LSC = large single copy region, SSC = small single copy region, IRA/B = inverted repeat A/B.

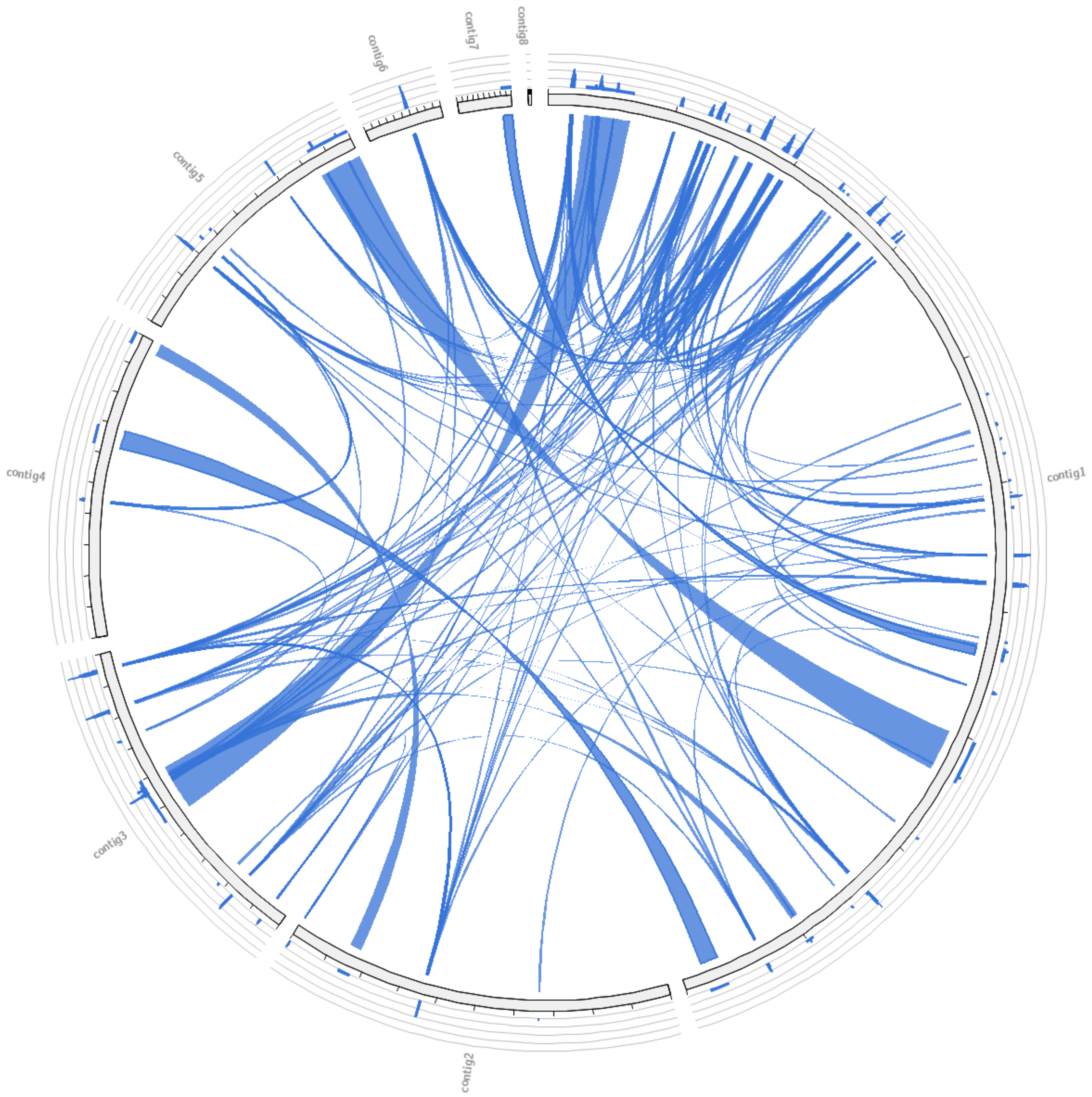
contig8
contig7
contig6
contig5
contig4
contig3
contig2
contig1

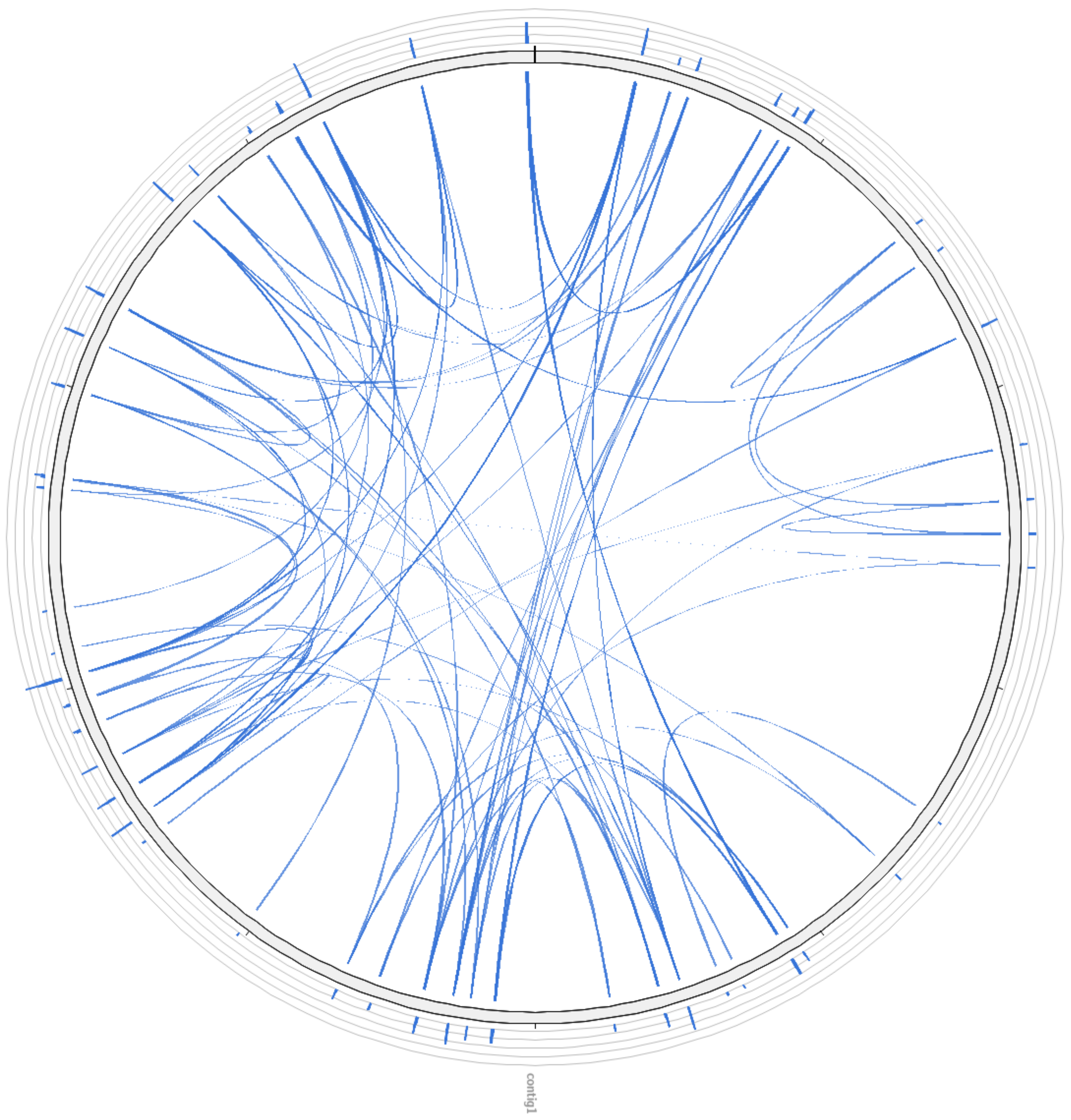
contig1

Fig. S3 (previous page). Dispersed repeats in the mitogenomes of *Cuscuta australis* (left) and *C. campestris* (right). Generated with Circoletto (http://tools.bat.infspire.org/circoletto/).

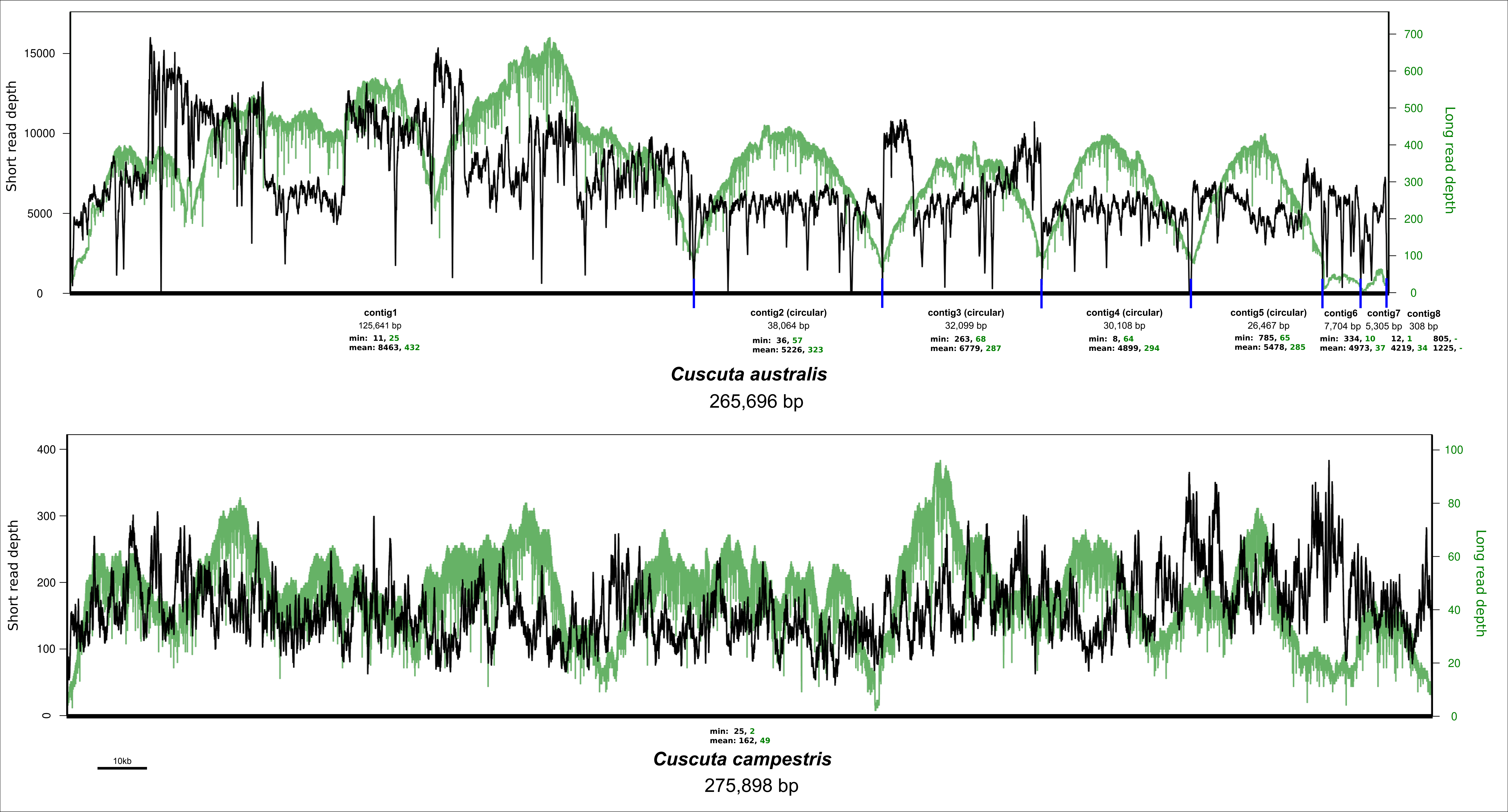

Short read depth
Long read depth
15000
10000
5000
0
700
600
500
400
300
200
100
0
contig1
125,641 bp
min: 11, 25
mean: 8463, 432
contig2 (circular)
38,064 bp
min: 36, 57
mean: 5226, 323
contig3 (circular)
32,099 bp
min: 263, 68
mean: 6779, 287
contig4 (circular)
30,108 bp
min: 8, 64
mean: 4899, 294
contig5 (circular)
26,467 bp
min: 785, 65
mean: 5478, 285
contig6
7,704 bp
min: 334, 10
mean: 4973, 37
contig7
5,305 bp
12, 1
4219, 34
contig8
308 bp
805, -
1225, -
Cuscuta australis
265,696 bp
400
300
200
100
0
100
80
60
40
20
0
min: 25, 2
mean: 162, 49
10kb
Cuscuta campestris
275,898 bp

Fig. S4 (previous page). Read depth for mitogenome assemblies of *Cuscuta australis* (top) and *C. campestris* (bottom). Short read depth is in black while long read depth is in green. Breakpoints between contigs in *C. australis* are blue lines. Minimum and mean read depths are indicated, with colours of text matching respective read depth.

Table S1. Repeats recombination analysis for *Cuscuta australis*. The analysis is based on non-overlapping repeats >500 bp and >90% identity (blastn evalue 1e-10), and mapping long reads to those repeats.

Repeat	Locus 1 contig	Locus 1 start	Locus 1 end	Locus 2 contig	Locus 2 start	Locus 2 end	Strand	Length (bp)	identity (%)	Reads spanning	# incongruous	% recombined	Note
1	3	14672	19276	1	3688	8292	-	4605	100.00	50	14	28	
2	5	22248	26467	1	88197	92416	-	4220	100.00	49	16	33	
3	4	18771	20608	1	121593	123430	-	1838	100.00	47	16	34	
4	4	28923	30108	2	31462	32647	-	1186	100.00	50	11	22	
5	7	4350	5305	1	79142	80097	-	956	100.00	50	N/A		hit is at end of contig (alternative path)
6	1	23546	24337	1	72459	73278	+	792	96.59	48	5	10	length locus 2 = 820
7	1	4229	4979	1	112453	113203	+	751	100.00	46	22	48	
8	1	15179	15726	1	125094	125641	+	548	100.00	50	N/A		hit is at end of contig (alternative path)
												**29**	**Average**

Table S2. Repeats recombination analysis for *Cuscuta campestris*. The analysis is based on non-overlapping repeats >500 bp and >90% identity (blastn evalue 1e-10), and mapping long reads to those repeats.

Repeat	Locus 1 start	Locus 1 end	Locus 2 start	Locus 2 end	Strand	Length (bp)	identity (%)	Reads spanning	# incongruous	% recombined	Note
1	16591	23986	214870	222265	+	7396	100.00	25	10	40	
2	37606	43001	64407	69802	+	5396	100.00	40	15	38	
3	220913	225483	260669	265239	+	4571	100.00	32	7	22	third alternative connection
4	22634	27068	111195	115629	+	4435	99.98	46	20	43	
5	108143	112547	257617	262021	+	4405	99.98	29	12	41	
6	138207	142395	271638	275898	+	4189	98.31	40	16	40	length locus 2 = 4261
7	164143	167027	198327	201209	+	2885	99.83	48	18	38	length locus 2 = 2883
8	71283	73704	101616	104037	+	2422	99.96	48	22	46	
9	58370	60787	246678	249126	+	2418	98.69	45	21	47	length locus 2 = 2449
10	192601	194502	253720	255621	+	1902	100.00	50	23	46	
11	81543	82685	183437	184579	+	1143	100.00	50	5	10	possible third alternative connection
12	129048	129774	162559	163285	+	727	100.00	49	16	33	
										37	Average all
										**41**	**Average two connections**

Tables S1 and S2 (previous page). Details of long repeats used for a proxy of recombinational activity in mitogenomes of *Cuscuta australis* and *C. campestris*. In two cases (highlighted) there were more than two alternative orientations inferred (the repeat occurs more than twice and involves connections to multiple contigs/locations in the genome), and in two cases in *C. australis*, the repeats hit the ends of contigs and could not be assessed.

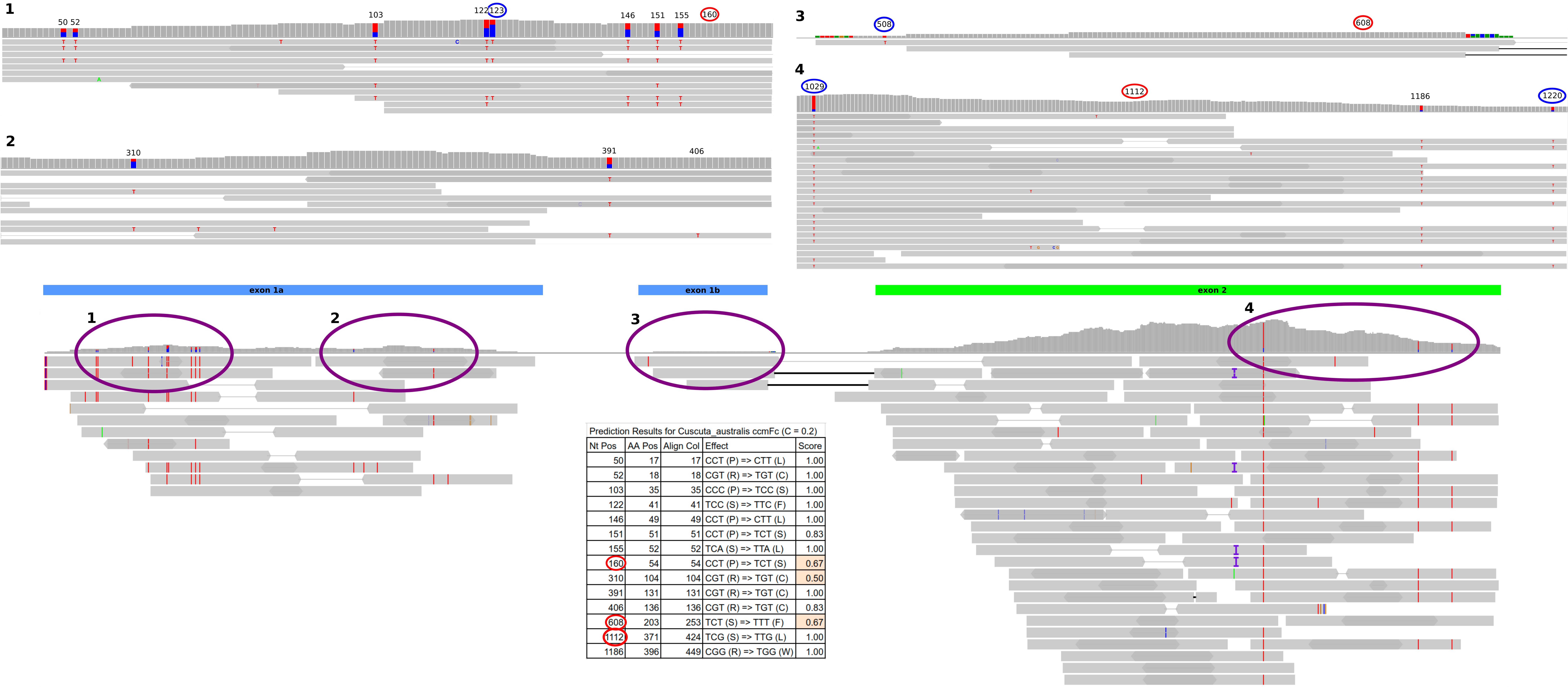

Prediction Results for Cuscuta_australis ccmFc (C = 0.2)

Nt Pos	AA Pos	Align Col	Effect	Score
50	17	17	CCT (P) => CTT (L)	1.00
52	18	18	CGT (R) => TGT (C)	1.00
103	35	35	CCC (P) => TCC (S)	1.00
122	41	41	TCC (S) => TTC (F)	1.00
146	49	49	CCT (P) => CTT (L)	1.00
151	51	51	CCT (P) => TCT (S)	0.83
155	52	52	TCA (S) => TTA (L)	1.00
160	54	54	CCT (P) => TCT (S)	0.67
310	104	104	CGT (R) => TGT (C)	0.50
391	131	131	CGT (R) => TGT (C)	1.00
406	136	136	CGT (R) => TGT (C)	0.83
608	203	253	TCT (S) => TTT (F)	0.67
1112	371	424	TCG (S) => TTG (L)	1.00
1186	396	449	CGG (R) => TGG (W)	1.00

Fig. S5 (previous page). Edited sites in *ccmF$_C$* from *Cuscuta australis* based on mapped transcriptomic reads and predicted by PREP-MT. Exons are indicated above the mapped reads, with circles corresponding to panels above showing the mismatches in more detail. Positions along the alignment are numbered, with positions not predicted by PREP-MT circled in blue, and those predicted but not observed by mapping circled in red. The table shows details of the results from PREP-MT.
