# Supplementary material for: Mitochondrial genomes of two parasitic Cuscuta species lack clear evidence of horizontal gene transfer and retain unusually fragmented ccmFC genes
Source: BMC Genomics. 2021 Nov 12;22:816. doi: 10.1186/s12864-021-08105-z (PMC8588681; doi:10.1186/s12864-021-08105-z)
Supplement: Supplementary file 4 — Additional file 4: Table S3. Reference plastomes used for annotation and BLASTN searches and their GenBank accession numbers. Table S4. Reference mitogenomes used for annotation, BLASTN searches and phylogenetic analysis and their GenBank accession numbers. [file 12864_2021_8105_MOESM4_ESM.pdf]

Additional file 4: supplementary Tables S3 and S4

Mitochondrial genomes of two parasitic *Cuscuta* species lack clear evidence of horizontal gene transfer and retain unusually fragmented *ccmF*$_C$ genes

Anderson, Benjamin M; Krause, Kirsten; and Petersen, Gitte

Table S3. Reference plastomes used for annotation and BLASTN searches and their GenBank accession numbers.

**Order**	**Family**	**Genus**	**Specific_ep**	**Accession number**
Asterales	Asteraceae	Helianthus	annuus	NC_007977.1
Boraginales	Lennoaceae	Pholisma	arenarium	NC_039719.1
Cucurbitales	Cucurbitaceae	Cucurbita	pepo	NC_038229.1
Ericales	Theaceae	Camellia	petelotii	NC_024661.
Fabales	Fabaceae	Glycine	max	NC_007942.1
Lamiales	Lamiaceae	Ocimum	basilicum	NC_035143.1
Magnoliales	Magnoliaceae	Liriodendron	tulipifera	NC_008326.1
Malvales	Malvaceae	Gossypium	barbadense	NC_008641.1
Poales	Poaceae	Oryza	sativa	NC_008155.1
Proteales	Nelumbonaceae	Nelumbo	nucifera	NC_025339.1
Santalales	Schoepfiaceae	Schoepfia	jasminodora	NC_034228.1
Solanales	Convolvulaceae	Cuscuta	exaltata	NC_009963.1
Solanales	Convolvulaceae	Cuscuta	obtusiflora	NC_009949.1
Vitales	Vitaceae	Vitis	vinifera	NC_007957.1

Table S4. Reference mitogenomes used for annotation, BLASTN searches and phylogenetic analysis and their GenBank accession numbers.

**Order**	**Family**	**Genus**	**Specific_ep**	**Accession number**
Alismatales	Araceae	Spirodela	polyrhiza	NC_017840.1
Alismatales	Butomaceae	Butomus	umbellatus	NC_021399.1
Alismatales	Hydrocharitaceae	Stratiotes	aloides	NC_035317.1
Alismatales	Zosteraceae	Zostera	marina	NC_035345.1
Amborellales	Amborellaceae	Amborella	trichopoda	KF754799.1–803.1
Apiales	Apiaceae	Bupleurum	falcatum	NC_035962.1
Apiales	Apiaceae	Daucus	carota	NC_017855.1
Aquifoliales	Aquifoliaceae	Ilex	pubescens	NC_045078.1
Arecales	Arecaceae	Cocos	nucifera	NC_031696.1
Arecales	Arecaceae	Phoenix	dactylifera	NC_016740.1
Asparagales	Amaryllidaceae	Allium	cepa	NC_030100.1
Asparagales	Orchidaceae	Gastrodia	elata	MF070084.1–102.1
Asterales	Asteraceae	Chrysanthemum	boreale	NC_039757.1
Asterales	Asteraceae	Diplostephium	hartwegii	NC_034354.1
Asterales	Asteraceae	Helianthus	annuus	NC_023337.1
Asterales	Asteraceae	Lactuca	saligna	NC_042406.1
Asterales	Asteraceae	Lactuca	sativa	NC_042756.1
Asterales	Asteraceae	Lactuca	serriola	NC_042378.1
Asterales	Asteraceae	Paraprenanthes	diversifolia	MN661146.1
Asterales	Campanulaceae	Codonopsis	lanceolata	NC_037949.1
Asterales	Campanulaceae	Platycodon	grandiflorus	NC_035958.1
Austrobaileyales	Schisandraceae	Schisandra	sphenanthera	NC_042758.1
Brassicales	Bataceae	Batis	maritima	NC_024429.1
Brassicales	Brassicaceae	Arabidopsis	thaliana	NC_037304.1
Brassicales	Brassicaceae	Arabis	alpina	NC_037070.1
Brassicales	Brassicaceae	Boechera	stricta	NC_042143.1
Brassicales	Brassicaceae	Brassica	carinata	NC_016120.1
Brassicales	Brassicaceae	Brassica	juncea	NC_016123.1
Brassicales	Brassicaceae	Brassica	napus	NC_008285.1
Brassicales	Brassicaceae	Brassica	nigra	NC_029182.1
Brassicales	Brassicaceae	Brassica	oleracea	NC_016118.1
Brassicales	Brassicaceae	Brassica	oxyrrhina	AP018041.1
Brassicales	Brassicaceae	Brassica	rapa	NC_016125.1
Brassicales	Brassicaceae	Capsella	rubella	NC_042883.1
Brassicales	Brassicaceae	Eruca	vesicaria	KF442616.1
Brassicales	Brassicaceae	Raphanus	sativus	NC_018551.1

Table S4 continued.

Order	Family	Genus	Specific_ep	Accession number
Brassicales	Brassicaceae	Schrenkiella	parvula	KT988071.2
Brassicales	Brassicaceae	Sinapis	arvensis	NC_031896.1
Brassicales	Brassicaceae	Turritis	glabra	LC325489.1
Brassicales	Caricaceae	Carica	papaya	NC_012116.1
Caryophyllales	Aizoaceae	Sesuvium	portulacastrum	MN683736.1
Caryophyllales	Caryophyllaceae	Silene	conica	JF750490.1–629.1
Caryophyllales	Caryophyllaceae	Silene	latifolia	NC_014487.1
Caryophyllales	Caryophyllaceae	Silene	noctiflora	JF750431.1–489.1
Caryophyllales	Caryophyllaceae	Silene	vulgaris	JF750427.1–30.1
Caryophyllales	Chenopodiaceae	Beta	macrocarpa	NC_015994.1
Caryophyllales	Chenopodiaceae	Beta	vulgaris	NC_002511.2
Caryophyllales	Chenopodiaceae	Chenopodium	quinoa	NC_041093.1
Caryophyllales	Chenopodiaceae	Spinacia	oleracea	NC_035618.1
Caryophyllales	Nepenthaceae	Nepenthes	ventricosa	NC_039531.1
Caryophyllales	Polygonaceae	Fallopia	multiflora	MF611850.1–1.1
Cucurbitales	Cucurbitaceae	Citrullus	lanatus	NC_014043.1
Cucurbitales	Cucurbitaceae	Cucumis	melo	MG947207.1–9.1
Cucurbitales	Cucurbitaceae	Cucumis	sativus	NC_016004.1–6.1
Cucurbitales	Cucurbitaceae	Cucurbita	pepo	NC_014050.1
Cycadales	Cycadaceae	Cycas	taitungensis	NC_010303.1
Ericales	Ericaceae	Monotropa	hypopitys	MK990822.1–3.1
Ericales	Ericaceae	Vaccinium	macrocarpon	NC_023338.1
Ericales	Theaceae	Camellia	sinensis	NC_043914.1
Fabales	Fabaceae	Acacia	ligulata	NC_040998.1
Fabales	Fabaceae	Ammopiptanthus	mongolicus	NC_039660.1
Fabales	Fabaceae	Ammopiptanthus	nanus	MH127920.1
Fabales	Fabaceae	Castanospermum	australe	MK426679.1
Fabales	Fabaceae	Cercis	canadensis	MN017226.1
Fabales	Fabaceae	Glycine	max	NC_020455.1
Fabales	Fabaceae	Glycine	soja	NC_039768.1
Fabales	Fabaceae	Haematoxylum	brasiletto	NC_045040.1
Fabales	Fabaceae	Leucaena	trichandra	NC_039738.1
Fabales	Fabaceae	Libidibia	coriaria	NC_045039.1
Fabales	Fabaceae	Lotus	japonicus	NC_016743.2
Fabales	Fabaceae	Medicago	truncatula	NC_029641.1
Fabales	Fabaceae	Millettia	pinnata	NC_016742.1
Fabales	Fabaceae	Phaseolus	vulgaris	NC_045135.1
Fabales	Fabaceae	Senna	occidentalis	NC_038221.1

Table S4 continued.

Order	Family	Genus	Specific_ep	Accession number
Fabales	Fabaceae	Senna	tora	NC_038053.1
Fabales	Fabaceae	Sophora	flavescens	NC_043897.1
Fabales	Fabaceae	Styphnolobium	japonicum	NC_039596.1
Fabales	Fabaceae	Tamarindus	indica	NC_045038.1
Fabales	Fabaceae	Vicia	faba	KC189947.1
Fabales	Fabaceae	Vigna	angularis	NC_021092.1
Fabales	Fabaceae	Vigna	radiata	NC_015121.1
Fabales	Polygalaceae	Epirixanthes	elongata	MG783394.1
Gentianales	Apocynaceae	Asclepias	syriaca	NC_022796.1
Gentianales	Apocynaceae	Cynanchum	wilfordii	MF611847.1–9.1
Gentianales	Apocynaceae	Rhazya	stricta	NC_024293.1
Geraniales	Geraniaceae	Geranium	maderense	NC_027000.1
Ginkgoales	Ginkgoaceae	Ginkgo	biloba	NC_027976.1
Lamiales	Gesneriaceae	Boea	hygrometrica	NC_016741.1
Lamiales	Gesneriaceae	Haberlea	rhodopensis	MH757117.1
Lamiales	Lamiaceae	Ajuga	reptans	NC_023103.1
Lamiales	Lamiaceae	Salvia	miltiorrhiza	NC_023209.1
Lamiales	Lentibulariaceae	Utricularia	reniformis	NC_034982.1
Lamiales	Oleaceae	Chionanthus	rupicola	MG372115.1
Lamiales	Oleaceae	Hesperelaea	palmeri	NC_031323.1
Lamiales	Oleaceae	Ligustrum	quihoui	MN723864.1
Lamiales	Oleaceae	Olea	europaea	MG372117.1
Lamiales	Orobanchaceae	Castilleja	paramensis	NC_031806.1
Lamiales	Phrymaceae	Mimulus	guttatus	NC_018041.1
Magnoliales	Magnoliaceae	Liriodendron	tulipifera	NC_021152.1
Malpighiales	Euphorbiaceae	Hevea	brasiliensis	AP014526.1
Malpighiales	Euphorbiaceae	Manihot	esculenta	NC_045136.1
Malpighiales	Euphorbiaceae	Ricinus	communis	NC_015141.1
Malpighiales	Salicaceae	Populus	alba	NC_041085.1
Malpighiales	Salicaceae	Populus	davidiana	NC_035157.1
Malpighiales	Salicaceae	Populus	tremula	NC_028096.1
Malpighiales	Salicaceae	Salix	purpurea	NC_029693.1
Malpighiales	Salicaceae	Salix	suchowensis	NC_029317.1
Malvales	Malvaceae	Bombax	ceiba	NC_038052.1
Malvales	Malvaceae	Corchorus	capsularis	NC_031359.1
Malvales	Malvaceae	Corchorus	olitorius	NC_031360.1
Malvales	Malvaceae	Gossypium	arboreum	NC_035073.1

Table S4 continued.

Order	Family	Genus	Specific_ep	Accession number
Malvales	Malvaceae	Gossypium	barbadense	NC_028254.1
Malvales	Malvaceae	Gossypium	davidsonii	NC_035075.1
Malvales	Malvaceae	Gossypium	harknessii	NC_027407.1
Malvales	Malvaceae	Gossypium	hirsutum	NC_027406.1
Malvales	Malvaceae	Gossypium	raimondii	NC_029998.1
Malvales	Malvaceae	Gossypium	thurberi	NC_035074.1
Malvales	Malvaceae	Gossypium	trilobum	NC_035076.1
Malvales	Malvaceae	Hibiscus	cannabinus	NC_035549.1
Myrtales	Lythraceae	Lagerstroemia	indica	NC_035616.1
Myrtales	Myrtaceae	Eucalyptus	grandis	NC_040010.1
Nymphaeales	Nymphaeaceae	Nymphaea	colorata	NC_037468.1
Pinales	Pinaceae	Pinus	Taeda	NC_039746.1
Poales	Poaceae	Aegilops	speltoides	NC_022666.1
Poales	Poaceae	Bambusa	oldhamii	EU365401.1
Poales	Poaceae	Cynodon	dactylon	MK175054.1
Poales	Poaceae	Eleusine	indica	NC_040989.1
Poales	Poaceae	Hordeum	vulgare	MN127975.1
Poales	Poaceae	Lolium	perenne	JX999996.1
Poales	Poaceae	Oryza	coarctata	MG429050.1
Poales	Poaceae	Oryza	minuta	NC_029816.1
Poales	Poaceae	Oryza	rufipogon	NC_013816.1
Poales	Poaceae	Oryza	sativa	NC_007886.1
Poales	Poaceae	Saccharum	officinarum	LC107874.1–5.1
Poales	Poaceae	Sorghum	bicolor	NC_008360.1
Poales	Poaceae	Tripsacum	dactyloides	NC_008362.1
Poales	Poaceae	Triticum	aestivum	NC_036024.1
Poales	Poaceae	Triticum	timopheevii	NC_022714.1
Poales	Poaceae	Zea	luxurians	NC_008333.1
Poales	Poaceae	Zea	mays	NC_007982.1
Poales	Poaceae	Zea	perennis	NC_008331.1
Proteales	Nelumbonaceae	Nelumbo	nucifera	NC_030753.1
Rosales	Cannabaceae	Cannabis	sativa	NC_029855.1
Rosales	Moraceae	Morus	notabilis	NC_041177.1
Rosales	Rhamnaceae	Ziziphus	jujuba	NC_029809.1
Rosales	Rosaceae	Eriobotrya	japonica	NC_045228.1
Rosales	Rosaceae	Malus	hupehensis	KR534606.1
Rosales	Rosaceae	Malus	x-domestica	NC_018554.1

Table S4 continued.

Order	Family	Genus	Specific_ep	Accession number
Rosales	Rosaceae	Prunus	avium	NC_044768.1
Rosales	Ulmaceae	Hemiptelea	davidii	MN061667.1
Santalales	Balanophoraceae	Lophophytum	mirabile	KU992322.1–80.1; KX792461.1
Santalales	Viscaceae	Viscum	album	NC_029039.1
Santalales	Viscaceae	Viscum	scurruloideum	KT022222.1–3.1
Sapindales	Anacardiaceae	Spondias	mombin	NC_045035.1
Sapindales	Anacardiaceae	Spondias	tuberosa	NC_045036.1
Sapindales	Nitrariaceae	Peganum	harmala	MK431826.1
Sapindales	Rutaceae	Citrus	sinensis	NC_037463.1
Sapindales	Sapindaceae	Xanthoceras	sorbifolium	MK333231.1
Saxifragales	Cynomoriaceae	Cynomorium	coccineum	KX270753.1–801.1
Solanales	Convolvulaceae	Ipomoea	nil	NC_031158.1
Solanales	Solanaceae	Capsicum	annuum	NC_024624.1
Solanales	Solanaceae	Hyoscyamus	niger	NC_026515.1
Solanales	Solanaceae	Nicotiana	attenuata	NC_036467.1
Solanales	Solanaceae	Nicotiana	sylvestris	NC_029805.1
Solanales	Solanaceae	Nicotiana	tabacum	NC_006581.1
Solanales	Solanaceae	Physochlaina	orientalis	NC_044153.1
Solanales	Solanaceae	Solanum	commersonii	MF989960.1–1.1
Solanales	Solanaceae	Solanum	lycopersicum	NC_035963.1
Solanales	Solanaceae	Solanum	pennellii	NC_035964.1
Solanales	Solanaceae	Solanum	tuberosum	MN104801.1–3.1
Vitales	Vitaceae	Vitis	vinifera	NC_012119.1
Welwitschiales	Welwitschiaceae	Welwitschia	mirabilis	NC_029130.1
